# Estimation of genetic parameters for hatching performance and genome-wide association analysis in Baicheng-You chickens

**DOI:** 10.3389/fvets.2026.1762660

**Published:** 2026-03-06

**Authors:** Gaoyun You, Haiying Li, Tinghao Jiang, Xiaoyu Zhao

**Affiliations:** College of Animal Science, Xinjiang Agricultural University, Ürümqi, Xinjiang, China

**Keywords:** Baicheng-You chickens, genetic parameter estimation, genome-wide association study, hatchability, whole-genome resequencing

## Abstract

The Baicheng-You chicken is a precious local chicken breed unique to Xinjiang, renowned for its strong stress resistance and excellent meat quality. However, its reproductive performance, particularly low hatching efficiency, severely restricts the industrial development of this breed. This study aims to systematically elucidate the genetic basis of the hatching performance of Baicheng-You chickens to provide a theoretical foundation for molecular breeding. A total of 844 44-week-old Baicheng-You chickens were studied, and key hatching traits such as the viable egg rate (VER), fertilization rate (FR), hatchability of eggs set (HES), hatchability of fertilized eggs (HFE), and chick hatching weight (CHW) were measured. High-density variation maps were constructed using whole-genome resequencing technology, genetic parameters were estimated using a mixed linear model, and genome-wide association studies (GWAS) were conducted to screen for significantly associated SNPs and candidate genes. Genetic parameter estimates revealed that the viable egg rate (VER, *h*^2^ = 0.27) and chick hatching weight (CHW, *h*^2^ = 0.40) exhibited moderate to high heritability, while the fertilization rate (FR, *h*^2^ = 0.07) and hatchability (HES, *h*^2^ = 0.02; HFE, *h*^2^ = 0.01) showed low heritability. GWAS identified 44 genome-wide significant SNPs (*p* < 4.36 × 10^−8^) and 130 suggestive significant SNPs (*p* < 8.71 × 10^−7^). Gene annotation identified 1,146 candidate genes significantly associated with the traits, including the *AvBD* family genes, *ATP5G3*, *JAM2*, *APP*, and *ABCE1*. Functional enrichment analysis indicated that these genes were significantly enriched in pathways closely related to embryonic immunity and developmental regulation, such as “defense response to bacterium,” “positive chemotaxis,” “cell cycle,” “ubiquitin-mediated proteolysis,” and the “apelin signaling pathway.” This study is the first to systematically reveal the genetic architecture of hatching performance in Baicheng-You chickens at the whole-genome level. The identified key genes and signaling pathways provide new insights into the molecular regulatory mechanisms of embryonic development and hatching efficiency. The findings not only offer important candidate targets for molecular marker-assisted selection in Baicheng-You chickens but also provide valuable genetic information and scientific support for the conservation and sustainable utilization of this unique local genetic resource.

## 1 Introduction

Poultry breeding industry serves as the cornerstone of modern animal husbandry development, with hatching performance being a key indicator for measuring the reproductive efficiency of breeding poultry. It directly influences the economic viability and sustainable development of farming operations. Traits such as viable egg rate, fertilization rate, and hatchability constitute the core biological bottlenecks in the transformation from breeding eggs to commercial generations. Deciphering the genetic mechanisms underlying these traits is of great significance for achieving precision breeding. However, current genetic research in poultry predominantly focuses on direct economic traits like egg production and growth rate. There is a lack of systematic exploration into the genetic basis of the critical developmental stage of incubation, particularly for local chicken breeds in China, where research remains relatively scarce. The Baicheng-You chicken, a distinctive local breed native to Xinjiang, has developed a unique genetic background through long-term natural and artificial selection. While widely recognized for its strong stress resistance and excellent meat quality, its reproductive performance—especially its low hatching efficiency—severely restricts the industrial utilization of this valuable genetic resource. Studies have shown that the hatchability of highly selected commercial breeds can reach 97.3% (1). In contrast, the hatchability of Baicheng-You chickens measured in this study was only approximately 80%, with prominent early embryonic mortality observed. This phenomenon suggests that hatching performance is likely regulated by specific genetic factors. Yet, no studies have systematically revealed its intrinsic mechanisms at the genomic level. In recent years, rapid advancements in genomic technologies have ushered in a new era for dissecting the genetics of complex traits. Genome-wide association studies (GWAS) have become a core method for identifying genetic loci associated with important traits, achieving significant progress in research on traits like egg production performance (2) and meat quality (3) in chickens. Studies on reproductive traits in local breeds, such as Wenchang chickens (4) and Chengkou Mountain chickens (5), provide valuable references for understanding the genetic diversity of avian reproduction. Nevertheless, existing research primarily concentrates on the egg-laying stage, with insufficient attention paid to the incubation process, which is a crucial link connecting genetic breeding and practical production. Embryonic development is a complex process involving the coordinated regulation of multiple gene networks (6). The efficiency of transforming a fertilized egg into a healthy chick is influenced by multiple factors, including maternal effects (7), embryonic vitality (8), and the intra-egg environment (9). Notably, several genes related to embryonic development discovered in recent chicken genomic studies, such as the *HOX* gene family (10) and *BMP* signaling pathway genes (11), may play important regulatory roles in hatchability traits. The critical functions of gene families like *PAX* (12) in mammalian embryonic development have been confirmed, but their roles in the avian incubation process remain unclear, offering new avenues for understanding the genetic basis of hatching performance. Concurrently, the successful application of genomic selection technology in animal husbandry demonstrates that genetic evaluation based on genome-wide markers can significantly improve breeding efficiency, providing technical feasibility for the genetic improvement of hatching performance in Baicheng-You chickens. This study utilizes whole-genome resequencing to systematically estimate genetic parameters and conduct GWAS for hatching performance in Baicheng-You chickens for the first time. By constructing a high-density genome-wide variation map, we aim to: (a) accurately estimate the genetic parameters of key hatching traits, including the viable egg rate (VER), fertilization rate (FR), hatchability of eggs set (HES), hatchability of fertile eggs (HFE), and chick hatching weight (CHW); (b) identify genetic loci and candidate genes significantly associated with hatching performance; and (c) reveal the genetic regulatory mechanisms affecting the embryonic development efficiency of Baicheng-You chickens. The research findings are expected to provide a theoretical basis for marker-assisted selection aimed at improving hatching performance in Baicheng-You chickens and to open new pathways for the conservation and efficient utilization of local chicken genetic resources.

## 2 Materials and methods

### 2.1 Experimental animals and phenotypic measurement

This study was conducted at the National Conservation Farm of Baicheng-You Chickens in the Aksu region of Xinjiang, China. A total of 844 healthy 44-week-old Baicheng-You hens with complete egg production records were included. All birds were housed individually in cages and fed a custom-formulated mash diet (Supplementary Table 1) during the laying period. Environmental conditions were strictly controlled, with temperature maintained at 20 ± 2 °C, relative humidity at 55–65%, and a photoperiod of 16 h light and 8 h dark. Artificial insemination was performed, commencing with four consecutive days of insemination. Egg collection started on the fourth day and continued for 10 days, with additional inseminations administered every 3 days throughout the collection period. The experimental protocol was approved by the Animal Ethics Committee of Xinjiang Agricultural University (Approval No. 2023007), and all procedures adhered strictly to animal welfare guidelines.

Hatching performance was evaluated based on the following key traits:

Viable Egg Rate (VER): The percentage of eggs produced that are suitable for incubation.


$$\mathrm{VER}(\%)=(\frac{\text{Number of viable eggs}}{\text{Total number of eggs produced}})\times 100\%$$

Fertilization Rate (FR): The percentage of incubated viable eggs that are confirmed as fertile via candling.


$$\mathrm{FR}(\%)=(\frac{\text{Number of fertile eggs}}{\text{Number of viable eggs}\;\mathrm{set}\;})\times 100\%$$

Hatchability of Eggs Set (HES): The percentage of all viable eggs set that hatched into live chicks, reflecting overall incubation efficiency and egg quality.


$$\mathrm{HES}(\%)=(\frac{\text{Total number of chicks hatched}}{\;\text{Number of viable eggs}\;\mathrm{set}\;})\times 100\%$$

Hatchability of Fertile Eggs (HFE): The percentage of fertile eggs that hatched successfully, directly indicating the inherent hatchability potential of fertilized eggs.


$$\mathrm{HFE}(\%)=(\frac{\text{Total number of chicks hatched}}{\text{Number of fertile eggs}})\times 100\%$$

Chick Hatching Weight (CHW): The individual body weight (in grams) of each chick measured within 24 h post-hatch using an electronic balance.

All trait data are presented as mean ± standard deviation. Prior to genetic analysis, raw phenotypic data underwent stringent quality control. Values beyond the range of mean ± 3 standard deviations were considered outliers and removed. Normality of the distribution for each trait was assessed using the Shapiro–Wilk test.

### 2.2 Blood sample collection and genomic DNA extraction

Whole blood samples were collected from all experimental chickens at 18 weeks of age. Genomic DNA was extracted using a commercial kit (Tiangen Biotech Co., Ltd., Beijing, China). DNA integrity was evaluated via 0.8% agarose gel electrophoresis, and the concentration was accurately measured using a Qubit 3.0 Fluorometer (Thermo Fisher Scientific Inc., Waltham, MA, USA). DNA samples with a total yield exceeding 1.5 μg were selected for library construction.

### 2.3 Reference genome alignment and quality control

Whole-genome resequencing reads were aligned to the bGalGal1.mat.broiler. GRCg7breference genome (Ensembl Release 110; https://ftp.ensembl.org/pub/release-110/fasta/gallus_gallus/dna/Gallus_gallus.bGalGal1.mat.broiler.GRCg7b.dna.toplevel.fa.gz) using BWA-MEM. Subsequent processing steps—including sorting, duplicate marking, and variant calling—were performed using the Sentieon pipeline (specifically, the util sort, Dedup, and Haplotyper modules, respectively). Based on the BAM files, Sentieon Haplotyper was run in GVCF mode for variant calling, generating per-sample variant files. The GVCFtyper module was then used to perform joint variant calling on the multi-sample GVCF files, producing a multi-sample VCF file. To ensure the accuracy of downstream genetic analyses, SNP data were subjected to strict quality control using PLINK (v1.90). Variant sites with a genotype missing rate exceeding 5% (--geno 0.05) and a minor allele frequency below 1% (--maf 0.01) were removed. Additionally, individuals with a sample missing rate higher than 20% (--mind 0.2) were filtered out.

### 2.4 Linkage disequilibrium analysis and data integration

To identify independent genetic loci, linkage disequilibrium (LD) pruning was performed using PLINK (v1.90) with the parameters --indep-pairwise 50 5 0.2 to remove redundant variants and enhance statistical power. As the genotype data subset was prepared, it was essential to ensure a complete and consistent match between the samples in the phenotype and genotype files for downstream genome-wide association study (GWAS). To guarantee data integrity and prevent the potential degradation of SNP quality due to sample size fluctuations, strict quality control was re-applied to the integrated dataset using PLINK (v1.90) with the parameters --geno 0.05 and --maf 0.01.

### 2.5 Principal component analysis

Principal component analysis (PCA) was performed on all autosomal SNPs using PLINK (v1.90). The first 20 principal components (PCs) were calculated for each individual, and PC1, PC2, and PC3 were plotted against each other for visualization. Significance testing of the principal components was conducted using EIGENSTRAT software (v6.1.4). The top 10 principal components were subsequently extracted and included as fixed-effect covariates in the genetic models to account for population stratification.

### 2.6 Phenotype preprocessing and genetic parameter estimation

Prior to genetic analyses, phenotypic data underwent stringent quality control. Values lying beyond the range of the mean ± 3 standard deviations were removed. Normality of distribution for each trait was assessed using the Shapiro–Wilk test. For phenotypes showing significant deviation from a normal distribution, rank-based inverse normal transformation was applied to meet the assumptions of parametric analysis.

Genetic parameters for the hatching traits were estimated using a linear mixed model within the ASReml-R package (v4.1.0.176). The model was built within the genomic best linear unbiased prediction (GBLUP) framework, utilizing a genomic relationship matrix (GRM) constructed from genome-wide markers to capture the aggregated effects of all SNPs for accurate heritability estimation. The model is specified as follows:


$$y=\mathrm{Xb}+Z_{1}a+Z_{2}c+e$$

Where:

*y* is the vector of observed phenotypes.

*X* is the design matrix for fixed effects (the final model included only the cage row (a categorical variable representing the physical location of the cage to account for environmental gradients in light, temperature, and ventilation) as a fixed effect to statistically control and correct for environmental variation caused by different physical positions, such as gradients in light intensity and temperature).

*b* is the solution vector for the fixed effect (cage row).

*Z*₁ and *Z*₂ are the design matrices for random effects.

*a* is the vector of additive genetic effects, assumed to be distributed as a ~ *N*(0, *Gσ*^2^*a*), where *G* is the genomic relationship matrix (GRM) constructed from genome-wide SNP data, and *σ*^2^*a* is the additive genetic variance.

*c* is the vector of permanent environmental effects, assumed to be distributed as *c* ~ *N*(0, *Iσ*^2^*c*), where *I* is an identity matrix, and *σ*^2^*c* is the variance component for the permanent environmental effect.

*e* is the vector of residual effects, assumed to be distributed as *e* ~ *N*(0, *Iσ*^2^*e*).

Genomic heritability (*h*^2^SNPs), representing the proportion of phenotypic variance explained by the cumulative effects of genome-wide SNPs, was calculated as:


h2SNPs=σ2a/(σ2a+σ2c+σ2e)

### 2.7 Genome-wide association analysis (GWAS)

Genome-wide association studies for hatching-related traits were performed using a mixed linear model (MLM) implemented in GEMMA software (version 0.96; https://github.com/genetics-statistics/GEMMA/releases, accessed June 17, 2025). The model was specified as follows to control for nongenetic variation:


y=Xα+Zβ+Wμ+ε

Where:

*y* is the vector of phenotypic values.

*X* is the design matrix for fixed effects, which included the cage position (as a proxy for environmental gradients in light and temperature) and the first ten principal components (PCs) to correct for potential population stratification.

α is the vector of estimated fixed-effect parameters.

*Z* is the design matrix for single nucleotide polymorphisms (SNPs).

β is the vector of SNP effects.

*W* is the design matrix for random effects.

μ is the vector of predicted random individual effects.

ε is the vector of random residuals.

To control for multiple testing, the genome-wide significance threshold was determined using Bonferroni correction. The effective number of independent tests (*N* = 1,147,912) was defined by the set of linkage disequilibrium (LD)-pruned SNPs (using PLINK with parameters: --indep-pairwise 50 5 0.2). Accordingly, the genome-wide and suggestive significance thresholds were set at *p* < 4.36 × 10^−8^ (0.05/*N*) and *p* < 8.71 × 10^−7^ (1/*N*), respectively.

### 2.8 Gene function annotation and enrichment analysis

Genotypes of SNPs that showed genome-wide significant associations with hatching traits were extracted. Phenotypic variation among different genotypes was assessed using appropriate non-parametric tests, specifically the Mann–Whitney *U* test and the Kruskal-Wallis test, as implemented in R. All analyses were anchored to the chicken GRCg7b reference genome assembly. Functional annotation of candidate SNPs was performed using the Variant Effect Predictor (VEP, Ensembl release 91, EMBL-EBI, Hinxton, UK). Genes located within 100 kb upstream and downstream of the lead SNP for each significant association were defined as candidate genes. To explore the biological functions of these candidate genes, Gene Ontology (GO) and Kyoto Encyclopedia of Genes and Genomes (KEGG) pathway enrichment analyses were conducted via the bioinformatics online platform^1^ (13). Significantly enriched terms and pathways were identified using the hypergeometric test, with the resulting *p*-values adjusted for multiple testing by controlling the false discovery rate (FDR) at a threshold of FDR < 0.05. In addition, to infer the potential biological relevance of significant SNPs located in non-coding regions, a co-localization analysis was performed by querying the chicken quantitative trait locus (QTL) data from the Animal QTLdb (Release 50). All reported QTLs overlapping the genomic intervals of the lead SNPs were retrieved, enabling the identification of known economic traits previously associated with these regions and thereby providing functional clues for the discovered variants.

### 2.9 Statistical fine-mapping and colocalization analysis

To identify potential causal variants within genome-wide significant loci, we performed statistical fine-mapping using the Sum of Single Effects (SuSiE) model. This Bayesian variable selection method, implemented in the R package susieR (version 0.14.2) (14), accounts for linkage disequilibrium (LD) structure and computes the posterior inclusion probability (PIP) for each variant, reflecting its likelihood of causality. A uniform prior was assumed, assigning equal initial probability of causality to all variants within a locus. Results are reported as 95% credible sets, defined as the smallest set of variants that contain at least one true causal variant with a posterior probability ≥95%. To investigate potential links between the GWAS signals and gene regulation, we further integrated the fine-mapping results with regulatory quantitative trait loci (QTL) data from the chicken Genotype-Tissue Expression (ChickenGTEx) project.^2^ Using a Bayesian colocalization framework (15), we evaluated the statistical evidence for a shared causal variant between the traits. A colocalization posterior probability (PPI) > 0.80 was set as the threshold for strong evidence of colocalization. Variants located within a fine-mapping credible set and showing colocalization with a regulatory QTL were prioritized as high-confidence candidate causal variants for subsequent analyses.

## 3 Results

### 3.1 Phenotypic statistics for hatching traits in Baicheng-You chickens

Descriptive statistics of the hatching traits in Baicheng-You chickens are presented in Table 1. After outlier removal, the effective sample size for each trait ranged from 827 to 844 individuals. As shown in Table 1, the mean fertilization rate (FR) and viable egg rate (VER) were 96.40 and 98.99%, respectively, indicating high overall values. In contrast, the mean hatchability of eggs set (HES) and hatchability of fertile eggs (HFE) were relatively low, at 76.51 and 81.23%, respectively. The average chick hatching weight (CHW) was 36.36 g. Based on the coefficients of variation (CV), HES (28.84%) and HFE (24.14%) exhibited substantial phenotypic variation within the population, suggesting a considerable degree of genetic variability and potential for genetic improvement through selection.

**Table 1:** Descriptive statistics of hatching traits in Baicheng-You chickens.

Traits	Count	Mean	Std	CV	Min	Max
FR (%)	827	96.40%	0.08	7.95%	62.50%	100.00%
VER (%)	844	98.99%	0.05	5.26%	40.00%	100.00%
HES (%)	844	76.51%	0.22	28.84%	12.50%	100.00%
HFE (%)	832	81.23%	0.20	24.14%	20.00%	100.00%
CHW (g)	837	36.36 g	2.89	7.97%	28.04 g	45.56 g

Count is the sample size, Mean is the average value, Std is the standard deviation, The coefficient of variation (CV) is defined as the ratio of the standard deviation to the mean, Min is the minimum value, Max is the maximum value.

### 3.2 Variant detection

To construct a high-quality genetic variation map for Baicheng-You chickens, this study performed whole-genome resequencing on 844 individuals at an average depth of 5×, ultimately yielding 2.37 Tb of raw data. Following alignment to the GRCg7b reference genome (alignment efficiency 99.73%) and quality assessment [Q30 score 93.26%; Supplementary Table 2(1–1)], the GATK pipeline was used for variant calling. From the initial detection of 28,597,909 raw variants (including 5,231,654 InDels and 23,366,255 SNPs), a stringent quality control process was applied, resulting in a high-quality core dataset comprising 1,931,564 SNPs (Supplementary Figure 1). This dataset was validated to be highly reliable, with a genotyping rate of 97.03%, a Ti/Tv ratio of 2.595, and a heterozygosity rate of 14.6% [Supplementary Table 2(1–2)], the data quality meets the requirements for subsequent analysis.

### 3.3 Estimation of genetic parameters

The estimation of genetic parameters for hatching traits in Baicheng-You chickens revealed a distinct genetic architecture (Table 2). Heritability estimates indicated that the fertilization rate (FR, *h*^2^ = 0.07), hatchability of eggs set (HES, *h*^2^ = 0.02), and hatchability of fertile eggs (HFE, *h*^2^ = 0.01) were lowly heritable. In contrast, the viable egg rate (VER, *h*^2^ = 0.27) exhibited moderate heritability, while chick hatching weight (CHW, *h*^2^ = 0.40) displayed a moderate-to-high heritability. Analysis of genetic and phenotypic correlations among the traits uncovered several key relationships. A very high positive genetic correlation (rg = 0.98) and a strong positive phenotypic correlation (rp = 0.86) were observed between HES and HFE. Simultaneously, HES also showed a strong positive genetic correlation with FR (rg = 0.93). Notably, a weak negative genetic correlation (rg = −0.25) was identified between CHW and FR, which may suggest an underlying physiological or genetic trade-off between chick weight at hatch and the rate of successful fertilization. These findings provide crucial parameters for deciphering the genetic architecture underlying hatching performance in Baicheng-You chickens and establish a theoretical foundation for informing future breeding strategies.

**Table 2:** Estimation of genetic parameters for hatching traits in Baicheng-You chickens.

Traits	FR	VER	HES	HFE	CHW
FR	0.07	−0.01	0.35^**^	0.02	−0.07^*^
VER	0.01	0.27	0.08^**^	0.07^**^	0.00
HES	0.93	0.78	0.02	0.86^**^	−0.15^**^
HFE	0.49	0.55	0.98	0.01	−0.13^**^
CHW	−0.25	−0.01	−0.56	−0.48	0.40

The diagonal represents the heritability estimates (*h*^2^); the off-diagonal elements below the diagonal represent the genetic correlations (rg), while those above the diagonal represent the phenotypic correlations (rp), the significance levels of the phenotypic correlation coefficients (above the diagonal) are indicated by asterisks: ^**^p < 0.01; ^*^p < 0.05.

### 3.4 Principal component analysis (PCA)

**Figure 1:**
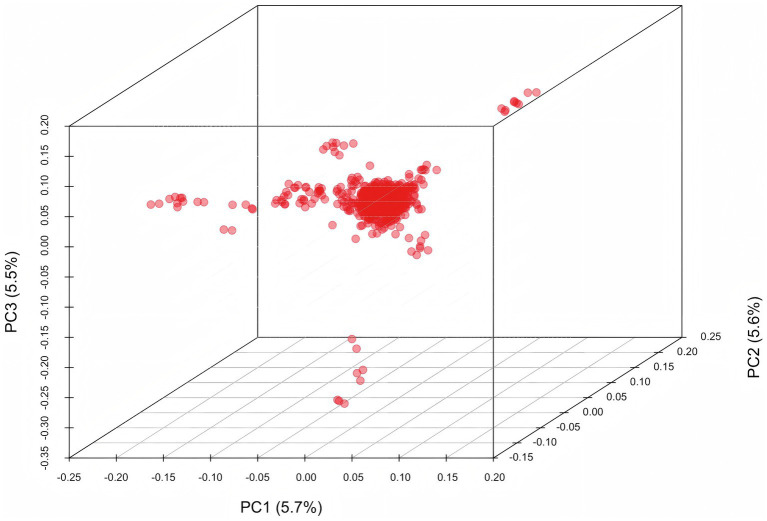
Scatter plot of principal component analysis. The plot illustrates the distribution of all individual samples (*n* = 844) in the three-dimensional space defined by the first three principal components (PC1, PC2, PC3) derived from high-density genome-wide single nucleotide polymorphism (SNP) data. Each red dot represents one individual.

Principal component analysis (PCA) was performed on a population of 844 Baicheng-You chickens. To account for potential confounding effects from population stratification, the top ten principal components were incorporated as fixed effects into the mixed linear model. The results (Figure 1) indicated no significant stratification pattern captured by the first three principal components.

### 3.5 Genome-wide association analysis of hatching performance

**Figure 2:**
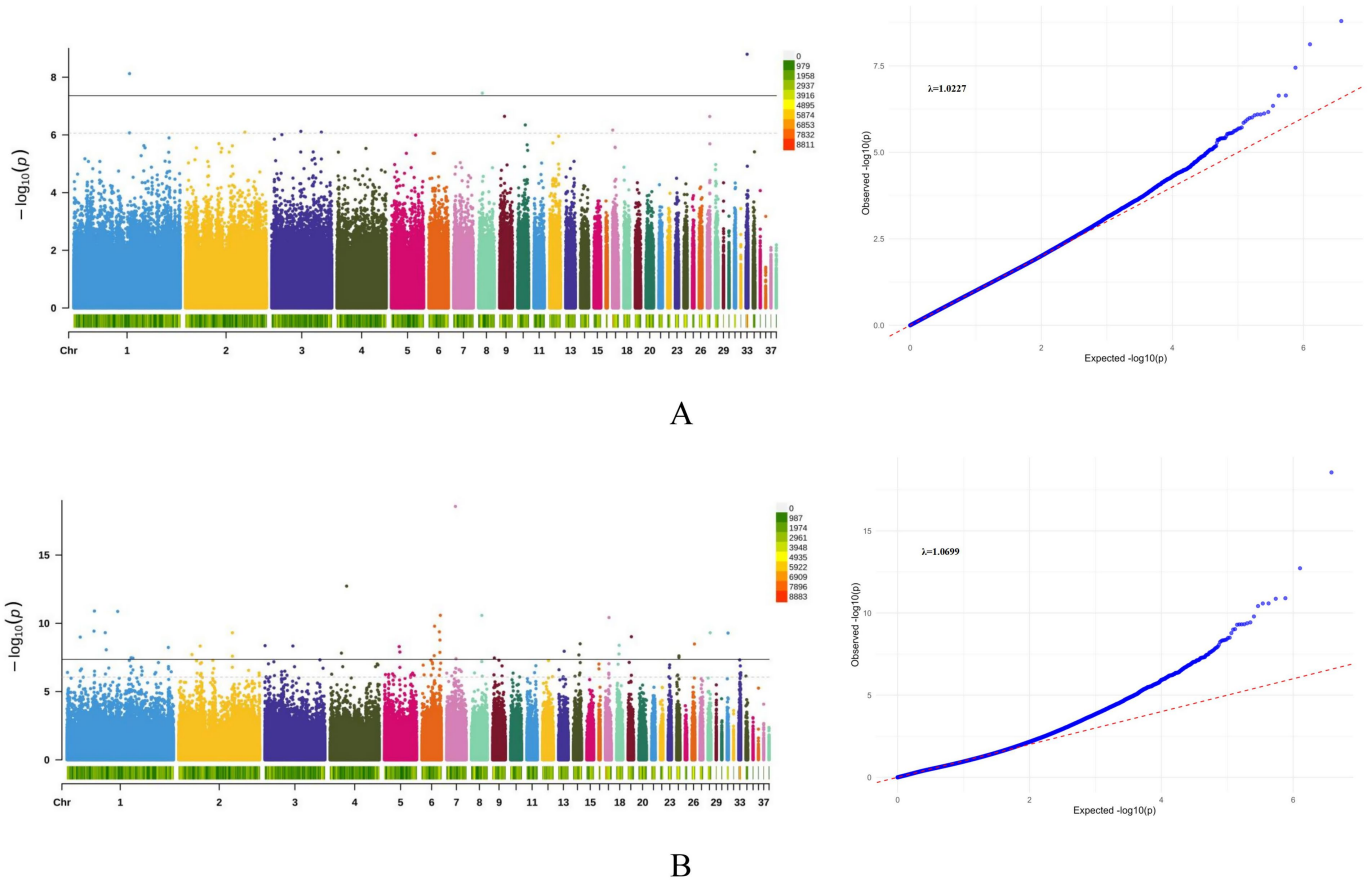
Manhattan and Q–Q plots of FR **(A)** and VER **(B)** in Baicheng-You chickens. Manhattan plots (left) and quantile-quantile (QQ) plots (right) display genome-wide association results for viable egg rate (VER; A) and fertilization rate (FR; B). The red dashed line indicates the genome-wide significance threshold, and the genomic inflation factor (*λ*) for each trait is shown.

Genome-wide association analysis for hatching performance identified 44 SNPs reaching genome-wide significance (*p* < 4.36 × 10^−8^), distributed across multiple chromosomes including GGA1-9, 13, 14, 17–19, 24, 26, 28, 31 and 33 (Table 3). Notably, the SNP 7:16293717 (Supplementary Table 6) exhibited the most pronounced effect on viable egg rate (VER), achieving genome-wide significance (*p* = 2.75 × 10^−19^) and explaining a high proportion of phenotypic variance (PVE = 9.12%). To ensure the robustness of the identified associations, we performed additional validation analyses for the lead SNP (7:16293717) associated with the viable egg rate (VER). Permutation testing (1,000 permutations) yielded an empirical *p*-value (EMP1) of 0.000999, confirming that the observed association is unlikely to be a false positive driven by population stratification or unaccounted confounding factors. Furthermore, genotype–phenotype analysis revealed a clear dose–response relationship: individuals with the TT genotype (*n* = 796) exhibited a VER of 2.85 ± 0.97, whereas the TC (*n* = 12) and CC (*n* = 5) genotypes showed progressively lower VER values (1.52 ± 2.17 and −0.95 ± 2.07, respectively). The apparent isolation of this significant peak in the Manhattan plot can be attributed to the low minor allele frequency (MAF ≈ 1.4%) and the low linkage disequilibrium (LD) in the genomic region, which is characteristic of true associations involving rare variants. These results collectively support the biological authenticity and statistical robustness of the identified SNP-trait associations. Gene annotation identified *ATP5G3* as the gene closest to this locus. Manhattan and Q–Q plots for fertilization rate (FR) and viable egg rate (VER) are shown in Figures 2A,B (see Supplementary Figure 2 for other traits), with genomic inflation factors (*λ*) of 1.0227 and 1.0699 respectively, indicating minimal population stratification and adequate confounding factors. Additionally, 130 suggestively significant SNPs were detected genome-wide, with 121 associated with VER, 8 with FR, and 1 with HFE (Supplementary Table 3). Functional annotation using VEP mapped these 174 significant and suggestive SNPs to 1,146 protein-coding genes (Supplementary Table 4), with approximately 8% located within gene regions and the majority in intergenic or regulatory regions. To explore the regulatory potential of intergenic variants, QTL analysis was performed on seven significant SNPs located within enhancer regions, revealing significant enrichment in QTL intervals for important economic traits (Supplementary Table 5). Specifically, SNPs 2:99120673, 17:6443612 and 1:50470803 showed strong colocalization with QTLs for egg quality and reproductive performance-traits directly influencing hatchability-providing critical clues and data support for future functional validation studies.

**Table 3:** Genome-wide significant SNPs associated with hatching performance traits in Baicheng-You chickens.

Traits	RS	ALT/REF	MAF	Beta ± SE	*p*_wald	PVE (%)
VER	1:24339663	G/A	0.013	−1.28 ± 0.21	1.01 × 10^−9^	4.33
VER	1:49731235	T/C	0.029	−0.89 ± 0.14	3.71 × 10^−10^	4.55
VER	1:50470803	A/G	0.028	−1.04 ± 0.15	1.26 × 10^−11^	5.30
VER	1:70439458	A/C	0.018	−1.10 ± 0.17	4.83 × 10^−10^	4.49
VER	1:72323703	A/T	0.064	−0.55 ± 0.10	8.70 × 10^−9^	3.85
VER	1:93432690	T/G	0.019	−1.15 ± 0.17	1.35 × 10^−11^	5.28
VER	1:118083684	T/C	0.023	−0.83 ± 0.15	3.35 × 10^−8^	3.55
VER	1:121585295	T/C	0.037	−0.68 ± 0.12	3.56 × 10^−8^	3.54
VER	1:186763208	C/G	0.063	−0.59 ± 0.10	5.83 × 10^−9^	3.94
VER	2:24423124	T/C	0.037	−0.70 ± 0.12	1.94 × 10^−8^	3.67
VER	2:39615284	T/C	0.014	−1.11 ± 0.19	4.60 × 10^−9^	3.99
VER	2:99120673	G/T	0.032	−0.74 ± 0.13	2.53 × 10^−8^	3.61
VER	2:99126174	A/G	0.028	−0.83 ± 0.13	4.94 × 10^−10^	4.49
VER	3:326311	A/T	0.013	−1.18 ± 0.20	4.33 × 10^−9^	4.01
VER	3:51555626	G/A	0.01	−1.23 ± 0.21	4.57 × 10^−9^	3.99
VER	4:21012717	C/T	0.012	−1.33 ± 0.23	1.52 × 10^−8^	3.73
VER	4:30603840	T/C	0.015	−1.26 ± 0.17	1.88 × 10^−13^	6.22
VER	5:27245342	A/G	0.013	−1.21 ± 0.20	5.09 × 10^−9^	3.97
VER	5:28407879	A/G	0.015	−1.06 ± 0.19	1.26 × 10^−8^	3.77
VER	6:22837927	A/G	0.035	−0.75 ± 0.13	2.33 × 10^−8^	3.63
VER	6:23519299	T/C	0.011	−1.35 ± 0.21	1.62 × 10^−10^	4.73
VER	6:32380531	G/A	0.013	−1.19 ± 0.19	4.20 × 10^−10^	4.52
VER	6:33268696	A/G	0.03	−0.80 ± 0.14	1.34 × 10^−8^	3.75
VER	6:33407920	C/T	0.037	−0.73 ± 0.12	1.67 × 10^−9^	4.22
VER	6:34190919	A/G	0.016	−1.24 ± 0.18	2.59 × 10^−11^	5.14
VER	7:16293717	T/C	0.014	−1.68 ± 0.18	2.75 × 10^−19^	9.12
VER	7:17409435	C/T	0.012	−1.18 ± 0.21	3.90 × 10^−8^	3.52
VER	8:19146243	A/G	0.014	−1.34 ± 0.20	2.61 × 10^−11^	5.13
VER	9:3305901	T/C	0.031	−0.76 ± 0.14	3.45 × 10^−8^	3.54
VER	13:10382255	G/C	0.016	−1.02 ± 0.18	1.11 × 10^−8^	3.80
VER	14:10088576	G/A	0.01	−1.27 ± 0.22	2.07 × 10^−8^	3.66
VER	14:12459945	T/C	0.014	−1.23 ± 0.21	3.16 × 10^−9^	4.08
VER	17:6671202	G/C	0.013	−1.23 ± 0.18	3.76 × 10^−11^	5.05
VER	18:4500874	T/C	0.013	−1.26 ± 0.22	1.76 × 10^−8^	3.69
VER	18:4889069	A/G	0.012	−1.12 ± 0.19	4.05 × 10^−9^	4.02
VER	19:6338807	C/T	0.013	−1.19 ± 0.19	9.54 × 10^−10^	4.34
VER	24:4231181	T/C	0.014	−1.10 ± 0.20	3.12 × 10^−8^	3.57
VER	24:4304515	A/G	0.038	−0.72 ± 0.13	2.43 × 10^−8^	3.62
VER	26:4494554	T/C	0.023	−1.01 ± 0.17	3.26 × 10^−9^	4.07
VER	28:3937247	A/G	0.011	−1.40 ± 0.22	4.89 × 10^−10^	4.49
VER	31:1723107	C/T	0.023	−1.08 ± 0.17	5.14 × 10^−10^	4.48
FR	1:102826107	C/T	0.026	−1.44 ± 0.25	7.51 × 10^−9^	3.88
FR	8:7181248	G/A	0.083	−0.78 ± 0.14	3.57 × 10^−8^	3.54
FR	33:2094390	G/A	0.015	−2.09 ± 0.34	1.60 × 10^−9^	4.23

This table presents single nucleotide polymorphisms (SNPs) that reached genome-wide significant levels in the genome-wide association study; RS: SNP identifier; ALT/REF: Effect allele/Reference allele; MAF: minor allele frequency; Beta ± SE: Effect size and its standard error; *p*_wald: *p*-value from the Wald test; PVE (%): Percentage of phenotypic variance explained by the SNP, PVE = [2 × MAF × (1 − MAF) × Beta^2^]/*σ*^2^*p*, where *σ*^2^*p* is the total phenotypic variance of the trait (Supplementary Table 8 for trait-specific *σ*^2^*p* values). The genome-wide significance threshold was set at *p* < 4.36 × 10^−8^, and the suggestive significance threshold was set at *p* < 8.71 × 10^−7^.

### 3.6 Functional enrichment analysis of candidate genes

**Figure 3:**
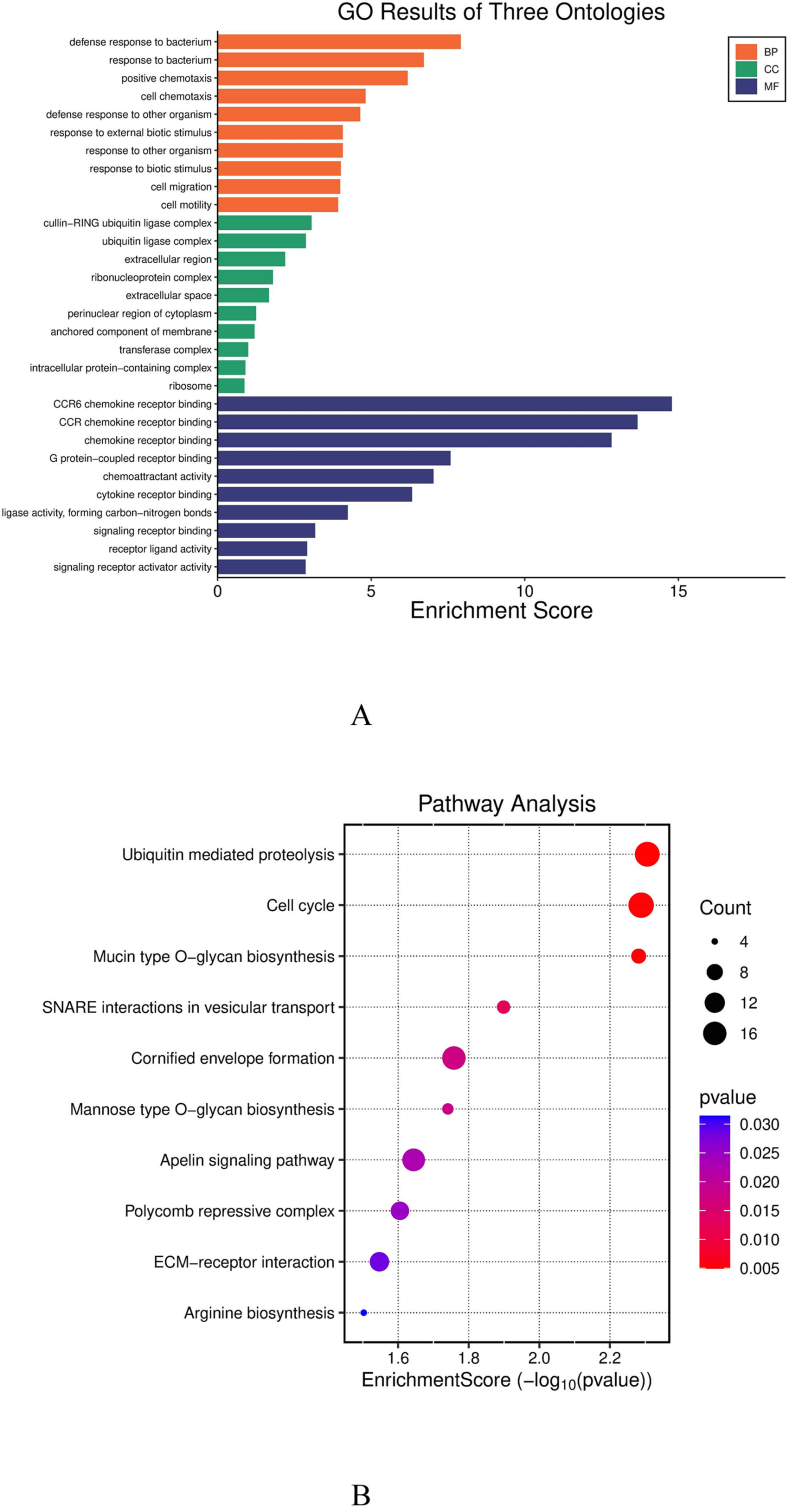
**(A)** GO enrichment pathway; **(B)** KEGG enrichment pathway. **(A)** Significantly enriched Gene Ontology (GO) terms are displayed, with bar length representing the statistical significance [−log_10_(*p*-value)] of enrichment across biological processes, cellular components, and molecular functions. **(B)** Significantly enriched Kyoto Encyclopedia of Genes and Genomes (KEGG) pathways are shown, where point size corresponds to the number of candidate genes mapped to each pathway, and color intensity indicates the level of enrichment significance.

A total of 1,146 protein-coding genes were screened by annotating the regions 100 kb upstream and downstream of the significant SNP loci, perform enrichment analysis on these genes (Supplementary Table 7). GO functional enrichment analysis (Figure 3A) revealed that these genes were significantly enriched in biological processes closely related to embryonic development, including “defense response to bacterium (GO:0042742),” “positive chemotaxis (GO:0050918)” and “CCR6 chemokine receptor binding (GO:0031731).” KEGG pathway analysis (Figure 3B) indicated that the candidate genes were significantly enriched in pathways such as “Ubiquitin mediated proteolysis (gga04120),” “Cell cycle (gga04110)” and “Apelin signaling pathway (gga04371).”

### 3.7 Fine-mapping analysis

**Figure 4:**
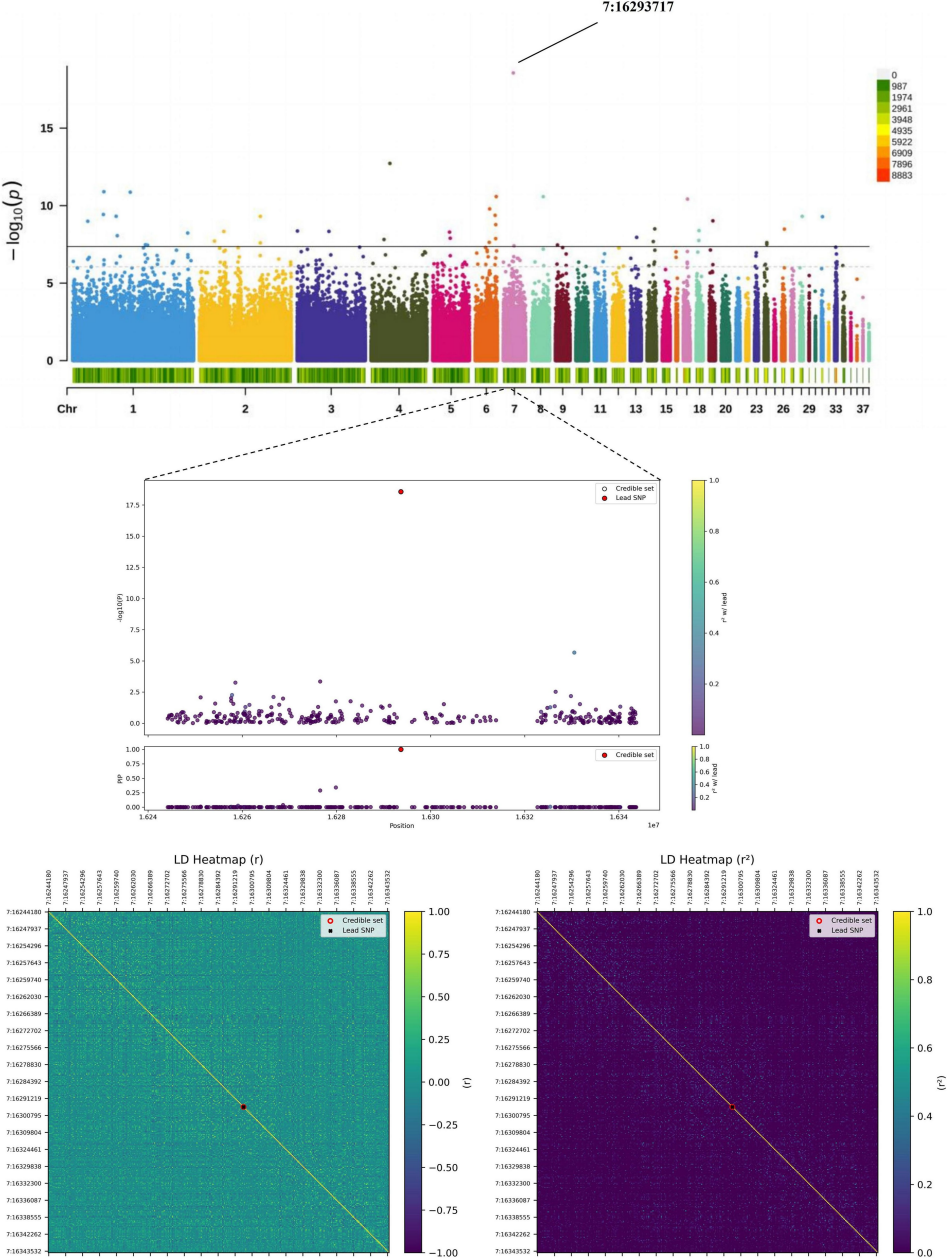
Fine-mapping of locus 7:16293717 for the VER trait. The top panel displays the regional association plot, with each point representing a SNP. The middle panel shows the statistical fine-mapping results. The bottom panels present linkage disequilibrium heatmaps based on *D*’ (left) and *r*^2^ (right) values relative to the lead SNP.

For the most significant loci identified in the genome-wide association analysis of fertilization rate (Supplementary Figure 3) and qualified egg rate, we performed fine-mapping analysis. As shown in Figure 4, this locus is significantly associated with the VER trait. The low degree of linkage disequilibrium in its surrounding regions strongly supports that chr7:16293717 itself is a potential causal variant rather than being linked to other loci. The 95% credible interval of this locus includes important genes such as *ATP5G3*, *GDF8*, and *LRP2*, which are key candidate genes for influencing hatching performance.

## 4 Discussion

The Baicheng-You chicken, a unique local genetic resource in China’s Xinjiang region, holds strategic significance for the protection and sustainable utilization of livestock and poultry genetic diversity in China. Currently, research on the reproductive traits of this breed, particularly a systematic genetic evaluation of hatching performance, remains a notable gap in the literature. This significantly hinders the efficient development and scientific breeding of this valuable resource. Therefore, as the first to elucidate the genetic basis of its hatching performance, this study addresses an urgent priority in poultry genetics and breeding research. This study, utilizing whole-genome resequencing, is to focus on the hatching traits of Baicheng-You chickens, aiming to fill this critical knowledge gap. This exploration will not only contribute to a fundamental understanding of the molecular mechanisms underlying its reproductive adaptability but also establish a solid foundation for developing a targeted marker-assisted breeding system and improving flock reproductive efficiency. Ultimately, it will facilitate the valorization and utilization of this distinctive genetic resource within modern animal husbandry.

The estimation of genetic parameters for key hatching traits in Baicheng-You chickens provides crucial insights into the genetic architecture of their reproductive performance. Our results indicate that the heritability of fertilization rate (FR), hatchability of eggs set (HES), and hatchability of fertile eggs (HFE) is low (*h*^2^ < 0.10), consistent with observations in most commercial chicken lines (16, 17). This suggests that these traits are strongly influenced by environmental, management, and non-genetic factors during embryonic development. In contrast, the viable egg rate (VER, *h*^2^ = 0.27) and chick hatching weight (CHW, *h*^2^ = 0.40) demonstrated moderate and moderate-to-high heritability, respectively, indicating a stronger genetic component and greater potential for improvement through genetic selection (18). Analysis of genetic correlations further revealed the underlying genetic structure. A very high positive genetic correlation was observed between HES and HFE (rg = 0.98), and both traits were strongly correlated with FR (rg = 0.93 and 0.49, respectively). This pattern suggests that the continuous developmental process from fertilization to successful hatching may be regulated by a common set of gene networks, supported by research on the genetic basis of embryonic survival (19). Notably, a moderate to high negative genetic correlation was found between CHW and hatchability traits (HES: rg = −0.56; HFE: rg = −0.48), implying that a higher hatching weight might increase energy demands during the embryonic stage, potentially negatively impacting survival under challenging conditions (20). Additionally, a weak negative correlation between CHW and FR (rg = −0.25) may suggest a genetic trade-off in parental reproductive investment strategy (21). In summary, these first systematic estimates of genetic parameters not only reveal the uniqueness of hatching performance in Baicheng-You chickens but also provide a key basis for formulating breeding strategies: greater emphasis on management optimization is needed for low-heritability traits, while a multi-trait selection index should be employed to balance medium-heritability traits exhibiting antagonistic relationships.

Based on the genome-wide association analysis (GWAS), we successfully identified 44 single nucleotide polymorphisms (SNPs) significantly associated with hatching performance at the genome-wide significance level (*p* < 4.36 × 10^−8^) in Baicheng-You chickens. The significant SNPs identified in this study underwent rigorous validation to preclude false positives. For instance, the lead SNP (7:16293717) for VER remained significant after permutation testing, and its genotype-dependent phenotypic gradient underscores a plausible biological effect rather than technical artifacts. The isolated appearance of this signal is consistent with a low-LD region and a rare variant, which often manifests as a single peak in Manhattan plots. These analyses reinforce the credibility of our GWAS findings and provide a solid foundation for subsequent fine-mapping and functional inference. Gene annotation of these significant loci identified candidate genes closely related to biological functions such as reproduction, development, and cellular energy metabolism, including *ATP5G3*, *ABCE1*, *JAM2*, *APP*, *OTUD4*, and *LOC121107942*. Here we primarily discuss *ATP5G3*, *ABCE1*, *JAM2*, and *APP*. *ATP5G3* encodes the c subunit of mitochondrial ATP synthase, which is a key component of the oxidative phosphorylation complex directly responsible for intracellular ATP production (22). Mitochondrial function is closely related to oocyte quality, as the developmental potential of oocytes is highly dependent on ATP provided by their mitochondria (23). In poultry, follicular development and egg formation are extremely energy-intensive processes. The most significant SNP annotated (7:16293717) is located near the ATP5G3 gene and explains a high proportion of phenotypic variance (PVE = 9.12%) for the viable egg rate (VER), indicating it is a major-effect locus. We hypothesize that genetic variation in *ATP5G3* may influence the energy metabolism efficiency of oviduct epithelial cells or follicular granulosa cells, thereby directly affecting key physical traits that determine VER, such as eggshell quality and membrane integrity. Some studies have indicated that mitochondrial dysfunction can lead to abnormal eggshell calcification (24). Furthermore, since all energy for embryonic development comes from nutrients stored in the egg and mitochondrial oxidative phosphorylation, the maternal *ATP5G3* genotype may also directly affect the energy metabolism efficiency and survival capability of the embryo itself. In broiler breeding, genes related to mitochondrial function have been demonstrated to associate with growth and efficiency traits (25, 26), but their role in reproductive traits, particularly hatching performance, requires further in-depth exploration. *JAM2* encodes junctional adhesion molecule 2, which is an important component of tight junctions and plays a central role in maintaining the integrity of tissue barriers such as the blood-testis barrier and blood–brain barrier (27, 28). In reproductive biology, the blood-testis barrier is essential for normal spermatogenesis, and its functional impairment directly leads to reduced FR (29). The significant association between *JAM2* and FR in this study strongly suggests that this gene may indirectly constrain population fertilization efficiency by influencing rooster sperm quality. More importantly, evidence indicates that *JAM2* also plays a significant role in early embryonic development. Research shows that *JAM2* exhibits a specific expression pattern in preimplantation mouse embryos, potentially involved in intercellular recognition and adhesion, thereby influencing embryonic developmental potential (30). In poultry, the oviduct serves as the key site for egg white and eggshell formation, and its internal environmental stability is crucial for early embryonic development (31). We speculate that *JAM2* may help maintain oviduct homeostasis, thereby providing a suitable early development microenvironment for the fertilized egg and directly affecting hatchability of eggs set (HES) and hatchability of fertile eggs (HFE). This hypothesis receives indirect support from several studies; for example, research in cattle suggests that the *JAM2*-rich microenvironment, created and maintained by the maternal reproductive tract under natural physiological conditions, may be crucial for early embryonic development and survival (32). Amyloid precursor protein (*APP*) is a transmembrane protein whose functions extend beyond its role in Alzheimer’s disease. *APP* plays important roles in various physiological processes including cell proliferation, adhesion, axonal guidance, and cell survival (33, 34). Studies show that *APP* is expressed in mammalian ovaries and oviducts and may participate in regulating reproductive tract physiological function (35). More critically, *APP* is considered a regulator of core embryonic development signaling pathways such as Wnt/β-catenin (36). In chicken embryos, the Wnt signaling pathway is vital for body axis formation and organ development, and pathway abnormalities can lead to early embryonic death (37). Therefore, we speculate that genetic variation in the *APP* gene may affect embryonic survival rates and thus associate with hatching performance by interfering with key developmental signaling pathways. ATP-binding cassette sub-family E member 1 (*ABCE1*) is an ATP-dependent ribosome recycling protein that plays a key role in translation termination, thereby globally regulating cell growth and proliferation (38). During the high-energy-demand stage of embryonic development, efficient protein synthesis mediated by *ABCE1* is fundamental for normal embryonic development. Research indicates that *ABCE1* is highly expressed in human and mouse oocytes and early embryos, and its functional inhibition severely compromises embryonic developmental potential (39, 40). Interestingly, a potential functional synergy may exist between *APP* and *ABCE1*: *APP* provides normal developmental signals and adhesion capacity for cells, while *ABCE1* supplies the protein synthesis foundation required for rapid embryonic development. This synergy suggests they may become key nodes affecting embryonic viability.

To gain deeper insights into the biological functions of genetic variants associated with hatching performance, we performed Gene Ontology (GO) and Kyoto Encyclopedia of Genes and Genomes (KEGG) enrichment analyses on the 1,146 candidate genes identified through our screening. The results revealed significant enrichment in multiple biological processes and pathways related to embryonic defense, immune regulation, and precise cellular control, providing important clues for elucidating the molecular regulatory mechanisms underlying the hatching performance of Baicheng-You chickens. In the GO analysis, the significant enrichment of terms such as “defense response to bacterium (GO:0042742)” suggests that the embryo’s innate immune system may play a critical role during the hatching process. Avian embryos develop within the enclosed environment of the eggshell, and their survival heavily relies on antimicrobial substances pre-existing in the egg white and yolk to defend against pathogenic microorganisms. Further analysis indicated that genes from the beta-defensin (*AvBD*) family occupy a central position within this functional module. Defensins, as a class of important antimicrobial peptides, serve as the first line of defense for the embryo against pathogens like bacteria and fungi. Studies have shown that *AvBD* genes are highly expressed in the uterus of the avian oviduct (the site of eggshell formation), and their products are directly deposited into the egg white and eggshell membranes, providing innate, passive immune protection for the embryo (41, 42). For instance, in chickens, genes such as *AvBD1*, *AvBD7*, and *AvBD9* have been confirmed to possess broad-spectrum antimicrobial activity (43–45). Genetic variations in this immune capacity may directly influence the hygienic status of the hatching eggs and the early survival rate of embryos. More importantly, evidence shows that certain *AvBD* members are not only involved in antimicrobial defense but also participate in regulating immune cell chemotaxis (consistent with the enrichment result for “positive chemotaxis, GO:0050918”) and the establishment of the early embryonic developmental microenvironment (46). For example, Jia et al. (47) found that polymorphisms in the chicken *AvBD10* gene were significantly associated with embryonic survival rates. Furthermore, research by Ayalew et al. (48) indicated that the expression levels of *AvBDs* in the embryonic liver change dynamically during development, suggesting their potential involvement in the development and activation of the embryo’s own immune system. Therefore, we speculate that genetic variations in the *AvBD* family genes in Baicheng-You chickens may collectively determine the embryo’s ability to resist infections by regulating the efficacy of antimicrobial substances within the egg and the maturation level of the embryonic immune system, thereby acting as key genetic factors affecting the hatchability of fertile eggs (HFE). In the KEGG pathway analysis, the significant enrichment of “Cell cycle (gga04110)” and “Ubiquitin mediated proteolysis (gga04120)” directs attention to the intrinsic life activities of embryonic cells themselves. Embryonic development is essentially a process of precise spatiotemporal regulation involving rapid cell proliferation, differentiation, and programmed cell death; dysregulation at any step could lead to developmental arrest or malformation. The cell cycle pathway ensures that cells undergo DNA replication and division at the correct time points, while the ubiquitination system precisely controls cell cycle progression, signal transduction, and DNA damage repair by degrading specific proteins (such as cyclins and misfolded proteins) (49, 50). In early chicken embryos, the function of cell cycle checkpoint proteins (e.g., CDC20) is crucial for maintaining genomic stability (51). Concurrently, the ubiquitin-proteasome system plays a core role in key developmental events such as the maternal-to-zygotic transition and axis formation (52). For example, aberrant expression of genes like Pramel15 can directly lead to early embryonic death in mice (53). Therefore, candidate genes enriched in these pathways, such as *ANAPC10* and *ANAPC13*, may help ensure genomic integrity and protein homeostasis during rapid embryonic cell proliferation, thereby clearing obstacles for normal embryonic development. The functional effectiveness of these genes is likely fundamental for achieving high hatchability. The enrichment of the “Apelin signaling pathway (gga04371)” offers an interesting perspective. Apelin is an important bioactive peptide that, upon binding to its receptor *APJ*, plays significant roles in regulating angiogenesis, energy metabolism, and fluid balance (54). In mammals, the Apelin signaling pathway has been confirmed to be crucial for the development of the embryonic cardiovascular system (55). For avian embryos, the establishment of the extra-embryonic circulatory system (particularly the chorioallantoic membrane vessels) is a prerequisite for gas exchange and nutrient acquisition, and its developmental integrity directly determines embryonic survival. Research shows that Apelin can promote the proliferation and migration of vascular endothelial cells (56). Furthermore, this pathway is also involved in regulating glucose metabolism and appetite, potentially influencing the energy utilization efficiency of the embryo (57). Studies in chicken embryos have shown that Apelin and its receptor are expressed as early as the initial stages of embryonic development (58–60). Consequently, we hypothesize that the Apelin signaling pathway might indirectly influence embryonic survival and developmental potential by coordinating the generation of the embryonic vascular system and energy metabolic homeostasis.

## 5 Conclusion

This study represents the first systematic investigation into estimating genetic parameters and performing a genome-wide association study for hatching performance in the Baicheng-You chicken, a distinctive local breed from Xinjiang, China. We successfully identified 174 SNPs associated with hatching performance that reached genome-wide or suggestive significance levels. An in-depth analysis of 1,146 protein-coding genes located within 100 kb upstream and downstream of these SNPs was conducted. Key genes, including *AvBD* family genes, *ATP5G3*, *JAM2*, *APP*, and *ABCE1*, were annotated as core candidate genes significantly associated with traits such as the viable egg rate and fertilization rate. These genes are thought to play important roles in mitochondrial energy metabolism, tissue barrier integrity, embryonic developmental signaling pathways, and the regulation of protein synthesis, thereby exerting a critical impact on hatchability. Gene Ontology (GO) and Kyoto Encyclopedia of Genes and Genomes (KEGG) pathway enrichment analyses revealed that these candidate genes are significantly enriched in biological processes and pathways closely related to embryonic immune defense, precise cellular regulation, and physiological function coordination, such as “defense response to bacterium,” “positive chemotaxis,” “Cell cycle,” “Ubiquitin mediated proteolysis” and the “Apelin signaling pathway.” Estimates of genetic parameters indicated that the viable egg rate and chick hatching weight exhibit moderate to high heritability, suggesting considerable potential for improvement through genetic selection. In contrast, fertilization rate and hatchability showed low heritability, indicating they may be more susceptible to environmental influences. These findings provide a new perspective for understanding the molecular regulatory mechanisms underlying hatching performance in Baicheng-You chickens. The results of this study not only offer a theoretical basis and candidate gene targets for marker-assisted selection aimed at improving hatching performance in this breed but also provide valuable genetic resources and scientific support for the conservation, selective breeding, and industrial development and utilization of this unique local chicken genetic resource.

## Data Availability

The variant data generated and reported from the genome-wide association analysis in this study have been deposited in the European Variation Archive (EVA) at EMBL-EBI under accession number PRJEB109041, https://www.ebi.ac.uk/eva/?eva-study=PRJEB109041.

## Footnotes

1
www.bioinformatics.com.cn

2
http://chicken.farmgtex.org
