# Supplementary material for: Estimation of genetic parameters for hatching performance and genome-wide association analysis in Baicheng-You chickens
Source: Front Vet Sci. 2026 Mar 6;13:1762660. doi: 10.3389/fvets.2026.1762660 (PMC13003602; doi:10.3389/fvets.2026.1762660)
Supplement: Supplementary file 9 [file Data_Sheet_1.PDF]

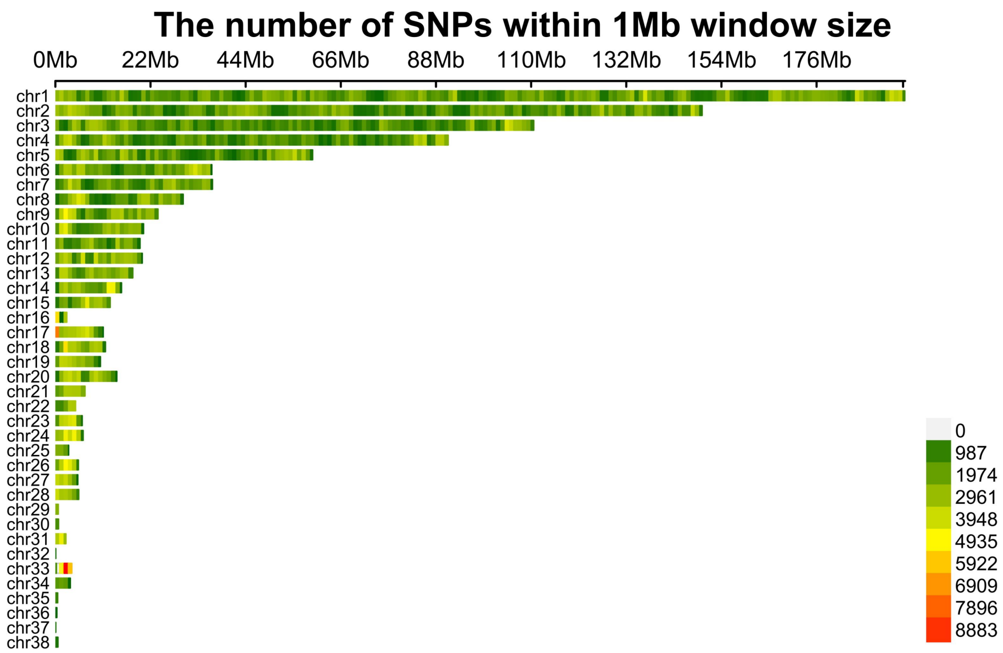

Supplementary Figure 1: SNP density map

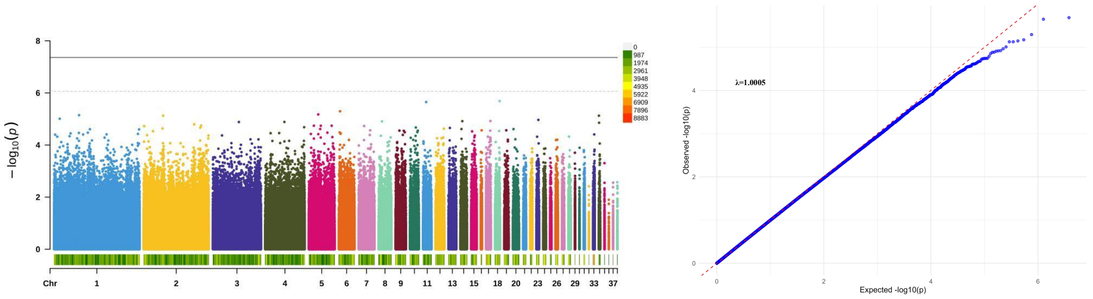

A

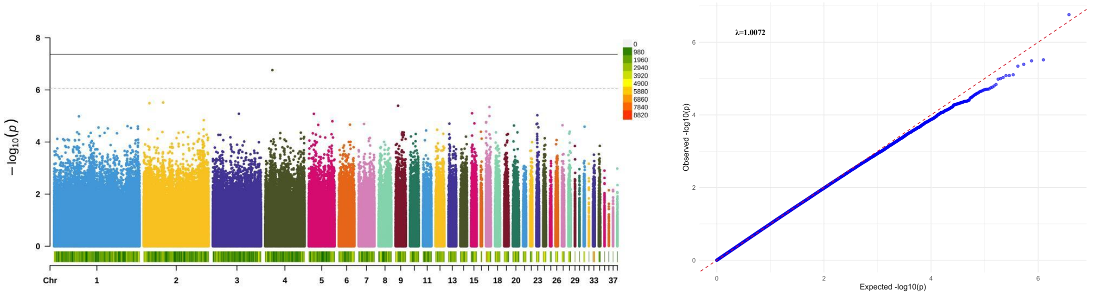

B

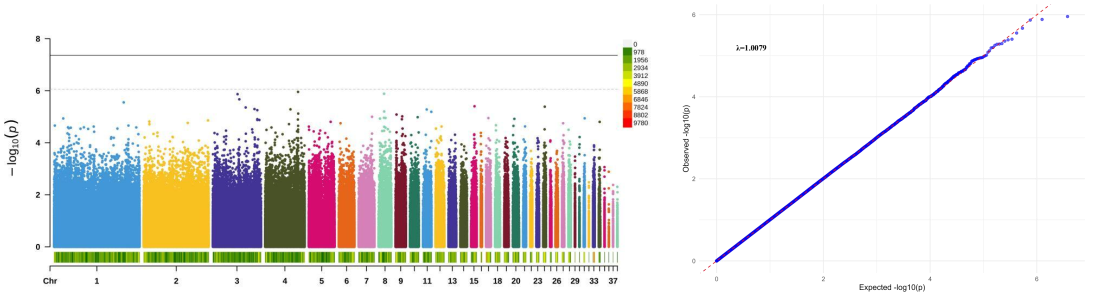

C

Supplementary Figure 2 (A): Manhattan plot and QQ plot of HES(Hatch of all eggs set); (B): Manhattan plot and QQ plot of HFE(hatching rate for fertilized eggs); (C): Manhattan plot and QQ plot of CHW(Chick hatch weight)

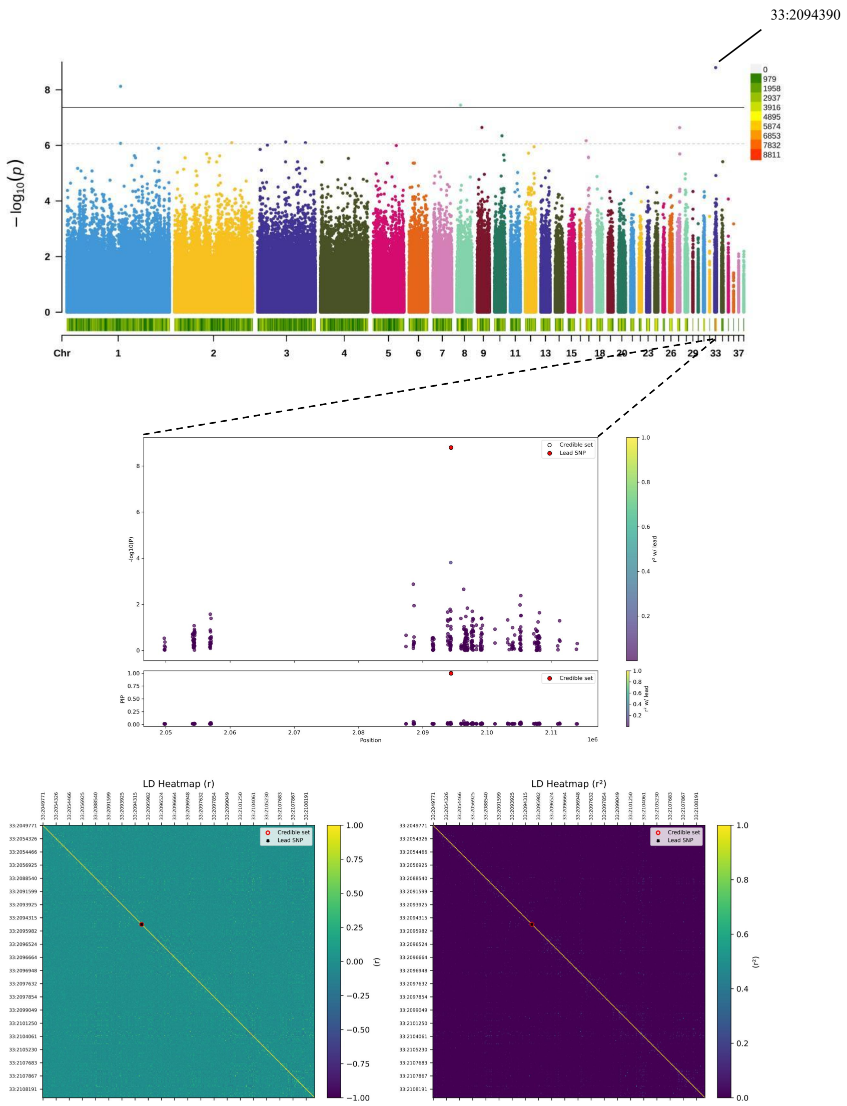
33:2094390
−log10(p)
Chr
0
979
1958
2937
3916
4895
5874
6853
7832
8811
Credible set
Lead SNP
-log10(P)
r² w/ lead
PIP
Position
1e6
LD Heatmap (r)
LD Heatmap (r²)

A

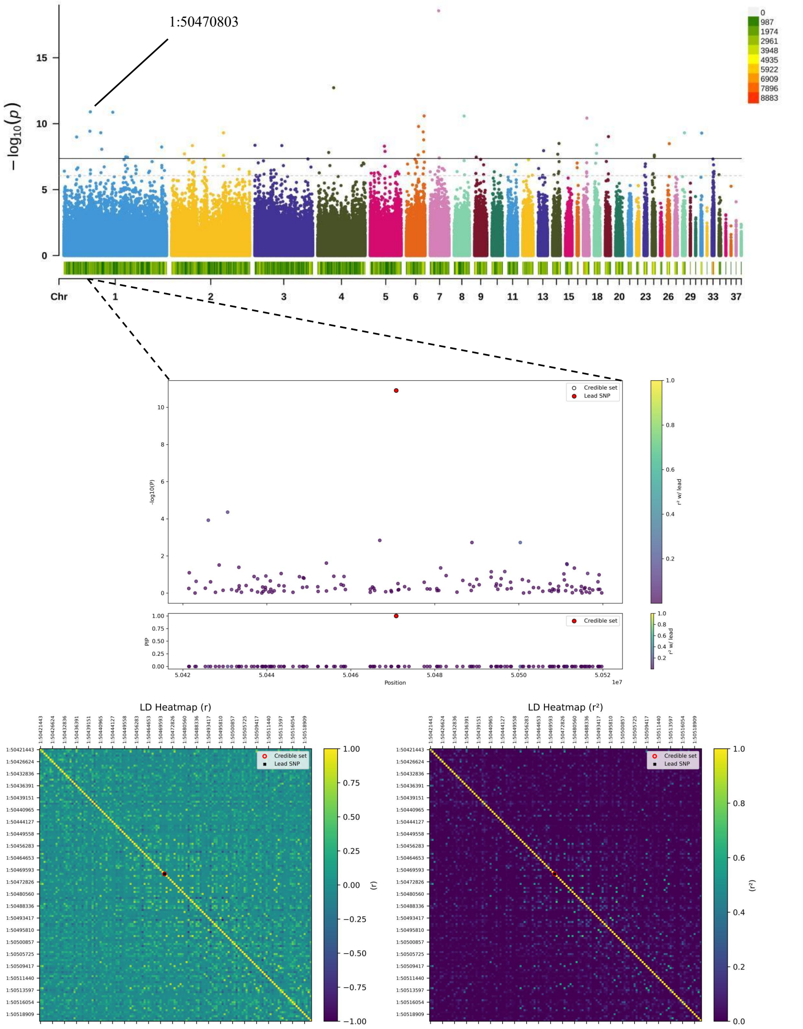

1:50470803
−log10(p)
Chr
Credible set
Lead SNP
Position
LD Heatmap (r)
LD Heatmap (r²)

B

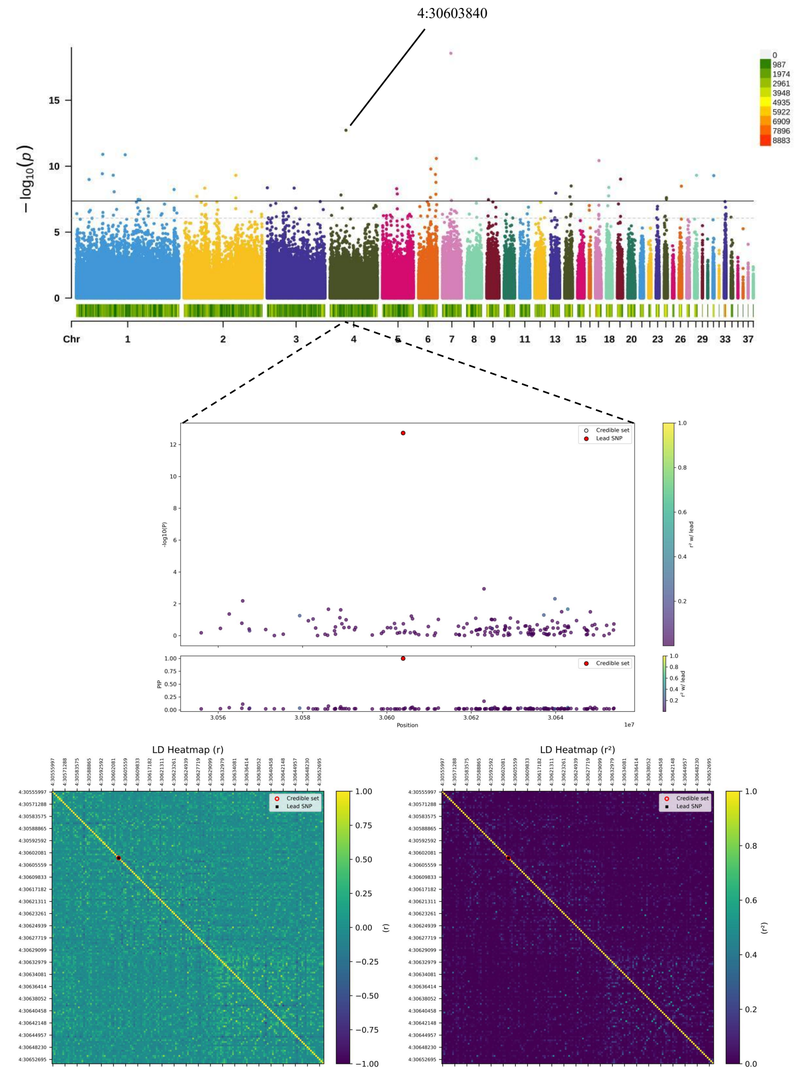

C

Supplementary Figure 3 (A): Fine-mapping of locus 33:2094390 for the FR trait; (B): Fine-mapping of locus 1:50470803 for the VER trait; (C): Fine-mapping of locus 4: 30603840 for the VER trait.
